# BAR Score Performance in Predicting Survival after Living Donor Liver Transplantation: A Single-Center Retrospective Study

**DOI:** 10.1155/2022/2877859

**Published:** 2022-02-18

**Authors:** Talaat Zakareya, Mohammad Taha, Hassan Elzohry, Ehab Darwiesh, Reda Aglan, Mostafa Elhelbawy, Hazem Zakaria, Mohamed Deif, Mohamed Abbasy

**Affiliations:** ^1^Hepatology and Gastroenterology Department, National Liver Institute, Menoufia University, Shebeen El-Kom, Egypt; ^2^Hepatopancreatobiliary and Liver Transplant Surgery Department, National Liver Institute, Menoufia University, Shebeen El-Kom, Egypt; ^3^Tropical Medicine Department, Faculty of Medicine, Zagazig University, Zagazig, Egypt; ^4^Radiology Department, National Liver Institute, Menoufia University, Shebeen El-Kom, Egypt

## Abstract

**Methods:**

146 adult liver transplant recipients were included. Univariate and multivariate analyses were used to determine the independent predictors of survival at 3 months, 1 year, and 5 years. The receiver operating characteristic (ROC) curve for the BAR score was plotted, and the area under the ROC curve (AUROC) was calculated. Kaplan–Meier curve and log-rank test were used to compare survival above and below the best cutoff values.

**Results:**

The mean age was 52.45 ± 8.54 years, and 59.6% were males. The survival rates were 89, 78.8, and 72% at 3 months, 1 year, and 5 years, respectively. The BAR score demonstrated a clinically significant value in the prediction of 3-month (AUROC = 0.89), 1-year (AUROC = 0.76), and 5-year survival (AUROC = 0.71). Among the investigated factors associated with survival, BAR score <10 points was the only independent predictor of 3-month (OR 7.34, *p* < 0.0001), 1-year (OR 3.37, *p*=0.001), and 5-year survival (OR 2.83, *p*=0.044).

**Conclusions:**

BAR is a simple and easily applicable scoring system that could significantly predict short- and long-term survival after LDLT. A large multicenter study is warranted to validate our results in the Egyptian population.

## 1. Introduction

Liver transplantation provides a curative treatment for most end-stage liver diseases. Given the increasing demand for liver transplantation, deceased organs are not sufficient enough to meet this increasing demand. Living donor liver transplantation (LDLT) provides an alternative to overcome this organ shortage [1].

Historically, the first liver transplantation in Egypt was performed in 1991 at the National Liver Institute, Menoufia University [2]. Since then, the liver transplantation program has expanded gradually over the past years. Currently, we have around 300 transplants performed at 13 centers across the country each year. For religious, cultural, and traditional beliefs, only LDLT is legalized by Egyptian law whereas deceased donor liver transplantation (DDLT) is prohibited [3]. Complications of hepatitis C virus (HCV) infection represent the leading indication for liver transplantation in Egypt. Several factors affect survival, including recipient, donor, surgical, and perioperative factors [4].

A scoring system that could precisely predict survival after LDLT is required to optimize the outcome. The model for end-stage liver disease (MELD) is a predictive model that was first used in 2000 to predict the survival of cirrhotic patients after transjugular intrahepatic portosystemic shunt operation [5]. Because of its yield in predicting short-term mortality in cirrhosis, it has been implemented in the United States and most western countries to prioritise liver transplant candidates on the waiting list since 2002. [6, 7].

In recent decades, some predictive models based on both donor and recipient data have been proposed to improve the ability to predict survival after liver transplantation. Rana et al. developed the Survival Outcomes Following Liver Transplantation (SOFT) score in 2008 [8]. The donor age-MELD (D-MELD) score was developed in 2009 as a product of incorporation of the recipient MELD at the time of transplantation and the donor age [9]. The balance of risk (BAR) score was developed by Dutkowski et al. in 2011 [10]. It was developed using the strongest six predictors of post-liver transplant survival derived from the data of 37255 patients in the UNOS (United Network for Organ Sharing) database: donor age (years), recipient age (years), cold ischemia time (hours), retransplantation (yes/no), life support (yes/no), and the MELD score at the time of liver transplant (true value without exception points). BAR score ranges from 0 to 27 points. The MELD score represents the greatest weight variable in the BAR score (0–14 points), followed by retransplantation (0 or 4 points), recipient age (0–3 points), life support (0 or 3 points), cold ischemia time (0–2 points), and donor age (0 or 1 point) [10].

Several studies have confirmed the superiority of BAR over other predictive systems such as donor risk index (DRI), MELD, D-MELD, and SOFT scores [11–15].

The BAR score is not used in nearly all liver transplantation centers in Egypt. This is because the BAR score was primarily developed and validated in Western countries, and there are no published data from Egypt to confirm its utility and validate its use in our population.

The goal of the present study is to evaluate the performance of the BAR score in the prediction of short- and long-term survival after LDLT in the Egyptian population.

## 2. Materials and Methods

### 2.1. Study Population

A retrospective observational cohort study was conducted on patients who underwent LDLT for different varieties of liver diseases at the liver transplantation center, National Liver Institute, Menoufia, Egypt, in the period between January 2008 and December 2016. All adult recipients (≥18 years) were enrolled. Pediatric liver transplants were excluded. Also, files with missing or incomplete data were excluded. The total number of liver transplants was 197. The number of pediatric transplants was 23, and the incomplete medical records were 28. The final eligible patients were 146. Demographic, clinical, laboratory, operative, and survival data (3 months, 1 year, and 5 years) for all eligible patients were collected. The BAR score was calculated online for all eligible patients. This is available at http://www.assessurgery.com/bar-score/bar-scorecalculator.

### 2.2. Statistical Methods

SPSS version 22.0 for Windows (IBM Corp, Armonk, NY, USA) was used for statistical analyses. Descriptive analysis of quantitative variables was expressed as mean and standard deviation (SD), while qualitative variables were presented as numbers and percentages.

Qualitative data were compared using the *χ*^2^ test or Fisher's exact test, where appropriate. A paired *t*-test or Wilcoxon rank test was used to compare continuous variables, while Mann–Whitney, Kruskal–Wallis, or Friedman tests were used to compare nonparametric data. A receiver operating characteristic (ROC) curve was plotted for the BAR score. The power of the BAR score to predict mortality at 3 months, 1 year, and 5 years was reflected by the area under the ROC curve (AUROC). It is widely accepted that the performance of a given diagnostic/prognostic test is adequate when AUROC is ≥ 0.7 while it is considered limited when AUROC is less than 0.7 [16, 17]. The best cutoff value was chosen using the Youden-J statistic (J = sensitivity + specificity – 1). Significant variables associated with survival in univariate analysis have been subjected to multivariate analysis using the Cox regression model. The Kaplan–Meier curve was used for survival analysis at the estimated time points (3 months, 1 year, and 5 years). The log-rank test was used to compare the survival of the groups below and above the chosen BAR cutoff value. The six variables contained in the BAR score were not included in the analysis. Graphical illustrations were created using the SPSS software. All analyses were considered statistically significant when the *p* value was less than 0.05.

## 3. Results

The mean age was 52.45 ± 8.54 years. Of those, 87 (59.6%) were males and 59 (40.4%) were females. The main underlying etiology of chronic liver disease was hepatitis C (85.6%). The rest of the patients' demographic, clinical, laboratory, and operative characteristics are shown in Table 1. The survival rates were 89, 78.8, and 72% at 3 months, 1 year, and 5 years, respectively.

### 3.1. Three-Month Survival Analysis

**Figure 1:**
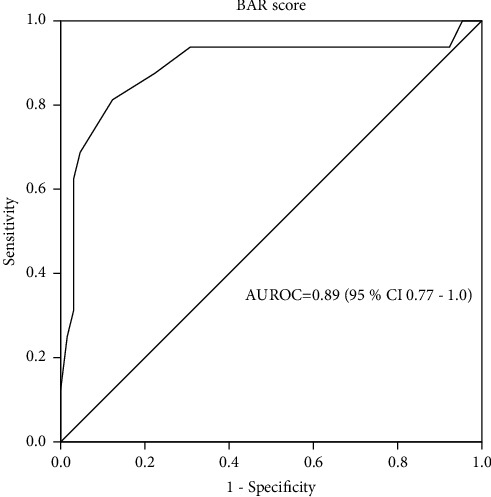
Receiver operating characteristic curve for the BAR score in predicting post-transplant 3-month survival. BAR, balance of risk; AUROC, area under the receiver operating characteristic curve.

Figure 1 represents the ROC curve analysis of the BAR score in predicting 3-month survival. The AUROC was 0.89 (95% CI 0.77–1.0, *p* < 0.001). Globally, an AUROC between 0.8 and 0.9 indicates an excellent prognostic yield. In view of this fact, the BAR score seems to be an accurate tool in predicting 3-month survival among our patients. The best cutoff value, as indicated by the Youden-J statistic, was 10, with a sensitivity of 87% and a specificity of 78%.

**Figure 2:**
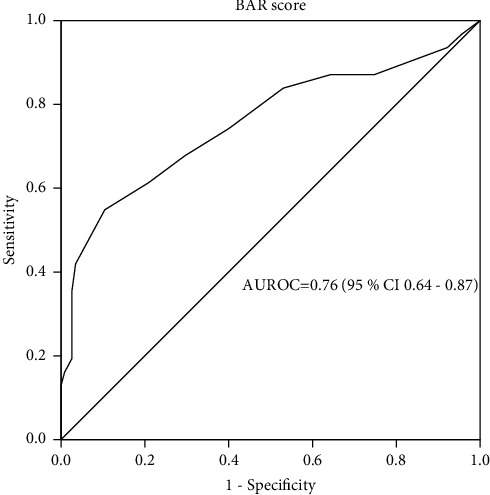
Receiver operating characteristic curve for the BAR score in predicting post-transplant 1-year survival. BAR, balance of risk; AUROC, area under the receiver operating characteristic curve.

In the univariate analysis, the significant variables associated with higher 3-month survival were a BAR score of less than 10 points (*p* 0.0001), an operative time of less than 12 hours (*p*=0.001), an intraoperative transfusion of less than 6 blood units (*p*=0.040), and an ICU stay of less than 10 days (*p*=0.046). In the multivariate analysis, the significant independent predictors of 3-month survival were only a BAR score of less than 10 points (OR 7.34, 95% CI 2.06–26.23, *p* < 0.0001) and a total operative time of less than 12 hours (OR 6.35, 95% CI 2.28–17.68, *p*=0.002) (Table 2).The Kaplan–Meier curve and the log-rank test (Figure 2) show a significantly higher 3-month survival in the group of patients with a BAR score of less than 10 points as compared with patients with a BAR score of ≥10 points (93.5% versus 65.2%, *χ*2 = 12.5, *p* < 0.0001).

### 3.2. One-Year Survival Analysis

**Figure 3:**
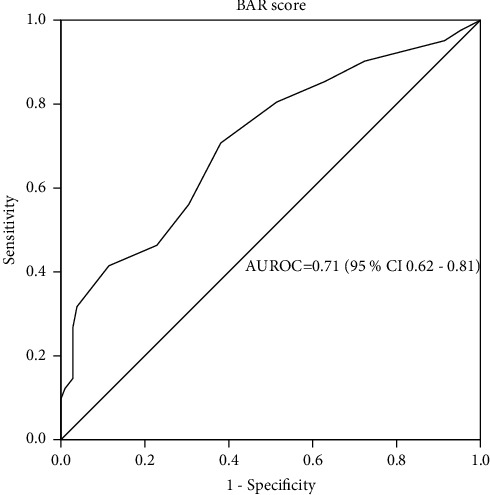
Receiver operating characteristic curve for the BAR score in predicting post-transplant 5-year survival. BAR, balance of risk; AUROC, area under the receiver operating characteristic curve.

As shown in Figure 3, the AUROC for predicting 1-year survival was 0.76 (95% CI 0.64–0.87, *p* < 0.001). An AUROC of ≥0.7 indicates a clinically useful prognostic test. So, the BAR score has adequate accuracy in predicting 1-year survival in our patients. The best-chosen BAR score cutoff value was 10, with a sensitivity of 62% and a specificity of 80%.

In the univariate analysis, the significant factors associated with higher 1-year survival were a BAR score of less than 10 points (*p*=0.001) and a total operative time of less than 12 hours (*p*=0.008). Both variables significantly predicted 1-year survival in multivariate analysis; a BAR score of less than 10 points (OR 3.37, 95% CI 1.46–7.08, *p*=0.001) and a total operative time of less than 12 hours (OR 3.21, 95% CI 1.6–7.2, *p*=0.004) (Table 2).

**Figure 4:**
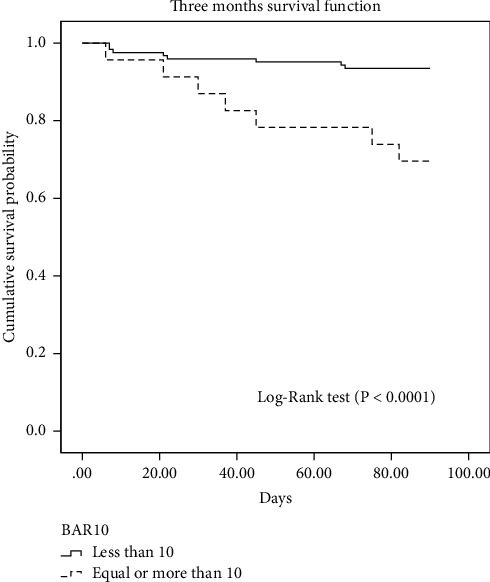
Post-transplant 3-month survival (Kaplan–Meier curve with log-rank test) for patients with the BAR score < and ≥10 points. BAR, balance of risk.

Figure 4 shows a comparison between 1-year survival in patients with a BAR score below and above 10 points. There was a significantly higher survival in the group of patients with a BAR score lower than 10 points (82.1% versus 60.9%, *χ*^2^ = 6.79, *p*=0.009).

### 3.3. Five-Year Survival Analysis

**Figure 5:**
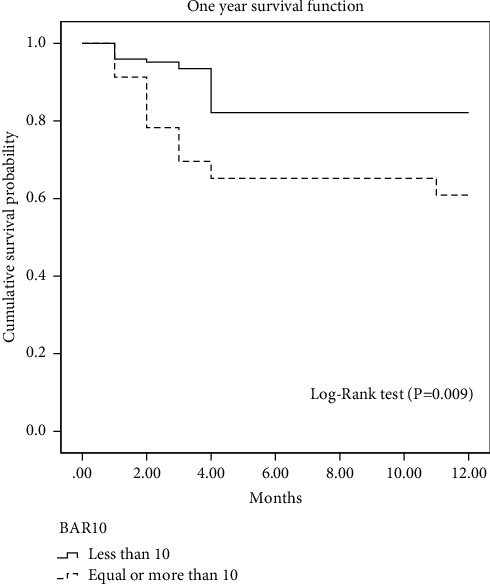
Post-transplant cumulative 1-year survival (Kaplan–Meier curve with log-rank test) for patients with the BAR score < and ≥10 points. BAR, balance of risk.

Figure 5 represents the ROC of the BAR score in predicting 5-year survival. The AUROC was 0.71 (95% CI 0.62–0.81, *p* < 0.001). This indicates that the BAR score is a statistically significant model with adequate accuracy in predicting 5-year survival in our population. The selected best cutoff value was 10, with a sensitivity of 56% and a specificity of 77%.

In the univariate analysis, the variables associated with a significantly higher 5-year survival were a BAR score of less than 10 points (*p* 0.002) and a total operative time of less than 12 hours (*p*=0.009). In multivariate analysis, the only significant independent predictor of 5-year survival was a BAR score of less than 10 points (OR 2.83, 95% CI 1.40–5.70, *p*=0.044) (Table 2).

**Figure 6:**
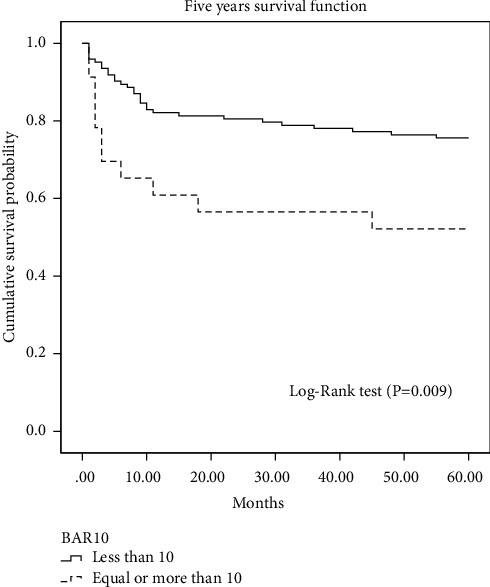
Post-transplant cumulative 5-year survival (Kaplan–Meier curve with log-rank test) for patients with the BAR score < and ≥10 points. BAR, balance of risk.

The Kaplan–Meier curve and the log-rank test (Figure 6) show a significantly higher 5-year survival in the group of patients with a BAR score of less than 10 points as compared with patients with a BAR score ≥10 points (75.6% versus 52.2%, *χ*^2^ = 6.81, *p*=0.009).

## 4. Discussion

Studying the predictors of survival after liver transplantation is essential for rationalising the use of resources and avoiding futile transplants. The ideal predictive model should match recipient, donor, and operative factors. In addition, it has to be simple and easy to apply. Several models have emerged with variable usefulness and limitations in clinical practice [15, 18–21]. The SOFT score is complex and not easily applicable as it includes 18 donor and recipient factors [8]. DRI has basically evolved to evaluate graft quality in cadaveric transplantation [9]. Although the MELD score accurately predicts waiting list mortality, its value in predicting post-transplant survival is limited [7, 22, 23]. When developed, the main target of D-MELD was to make a good donor-recipient match in the setting of DDLT. Its basic limitation in LDLT is the negligence of other fundamental operative and perioperative factors that undoubtedly affect the survival outcome. Furthermore, Egyptian law limited the donation age to between 21 and 45 years. In real life, more than 80% of our donors were aged between 21 and 30 years. This could reduce the weight of the age factor in the D-MELD score [3]. The BAR score fulfils most of the proposed criteria of the ideal predictive model, and its utility has been confirmed in the prediction of post-liver transplantation survival outcomes [11–15, 24].

To our knowledge, this is the first study to evaluate the value of the BAR score in predicting survival after liver transplantation in the Egyptian population.

The current study demonstrated that the BAR score is excellent in predicting 3-month survival after liver transplantation.

The AUROC was 0.89 (95% CI 0.77–1.0, *p* < 0.001). In the study by Boecker et al., AUROC was 0.847(CI 0.761–0.934; *p* < 0.001) for the prediction of 90-day mortality [21]. This robust performance of the BAR score has been confirmed in the study by Conjeevaram et al. where the AUROC was 0.80 (CI 0.73–0.88) [15]. Martínez et al. reported similar results in the analysis of 3-month survival; AUROC was 0.755 (95% CI 0.689–0.812) [25].

On the other hand, other studies failed to confirm reasonable accuracy for the BAR score where AUROC came below 0.7 [12, 13, 18, 26]. In the multivariate analysis, a BAR score of less than 10 points was an independent predictor of post-transplant 3-month survival. The survival rates below and above a BAR score of 10 points were 93.5% and 65.2%, respectively (*p* < 0.0001). Boecker et al. [21] reported significantly lower 90-day mortality in patients with a BAR score of less than 14 points (2.3% versus 22.2%, respectively; OR 11.857, CI 4.441–31.657, *p* < 0.001). Similarly, Martinez et al. [25] reported that patients with a BAR score of less than 15 points had a significantly higher 3-month survival (93% versus 67%, respectively; Hazard ratio 0.210, 95% CI 0.078–0.562, *p* 0.01). Our univariate analysis revealed that the number of blood units transfused during the surgery was a statistically significant predictor of 3-month survival (*p*=0.04). However, this finding could not be confirmed in the multivariate analysis. This came in concordance with what was reported by Martínez et al. [25]. Other authors, on the other hand, found a significant association between the amount of transfused blood and short-term mortality after liver transplantation [13, 27, 28]. Also, the ICU stay was statistically significant in the univariate analysis (*p*=0.046), yet it became insignificant in the multivariate analysis. In the study by Stratigopoulou et al., the mean survival of patients with a prolonged ICU stay was significantly lower than that of those discharged from the ICU within 3 days after transplantation surgery (69.057 months, 95% CI 62.402–75.711 versus 87.943 months, 95% CI 81.162–94.724, log-rank test 14.088, *p* < 0.001) [29].

Our results revealed that the BAR score performed well in predicting 1-year survival (AUROC = 0.76, 95 % CI 0.64–0.87, *p* < 0.001). This came in consonance with the findings reported by Martinez et al. (AUROC 0.702, 95% CI 0.634–0.764) [25]. The survival rate was significantly higher in the group of patients with a BAR score of less than 10 as compared with those with a BAR score ≥10 (82.1% versus 60.9%, *p*=0.009). Similar findings were reported by Torterolli et al. [26] where one-year survival for patients with a BAR score ≤9 was 73.9% versus 51.6% with a BAR >9 (*p*=0.001). Also, Martinez et al. have documented a significantly higher one-year survival in patients with a BAR score of less than 15 points than those with a BAR ≥15 points (88% versus 59%, *p* < 0.01) [25].

A total operative time of less than 12 hours was found to independently predict 3-month and 1-year post-transplant survival. This is matched with the findings of Stratigopoulou et al. who reported that prolonged duration of transplant surgery is significantly associated with postoperative complications, prolonged stay in the ICU, and hence increased short-term mortality and reduced overall survival [29].

In continuity of its role in predicting 3-month and 1-year survival, the BAR score showed an adequate accuracy in predicting 5-year survival where AUROC was 0.71 (95% CI 0.62–0.81, *p* < 0.001). This contradicted the findings of Martinez et al., who found that the clinically useful threshold for AUROC could not be reached (AUROC = 0.61) [25]. Likewise, de Boer et al. reported a limited value of the BAR score in predicting post-transplant 5-year survival (c-statistic = 0.56) [20].

In the current study, the cumulative 5-year survival was significantly higher in patients with a BAR score of less than 10 points as compared with those with a BAR score ≥10 points (75.6% versus 52.2%, *p*=0.009). This is concordant to what was reported by Boecker et al. where the five-year survival rate was 76% for BAR ≤14 versus 69% for BAR >14 (*p*=0.042) [21].

Notably, the BAR score cutoff values were variable among different studies. The selected cutoff value in our cohort was 10 points. The cutoff value used in the current study (10 points) was quite similar to the cutoff value used by Torterolli et al. (9 points). This value, on the other hand, is lower than the cutoffs used by other authors. For example, Boecker et al. used 14 points, Martinez et al. used 15 points, and Dutkowski et al. used 18 points as the best cutoff values [10, 21, 25]. This difference could be explained by the variability in the sample size and the relatively younger donors (26.45 ± 6.06 years) in our cohort. In addition, the mean MELD score, which is a highly weighted component of the BAR score, was lower in the present study as compared with other studies. Furthermore, none of our patients received more than one transplant. For these reasons, the BAR score values in the present study seem to be relatively lower than in other studies.

It has to be noted that using a high cutoff value would be beneficial in deciding the futility of transplantation as reported by Dutkowski et al., who considered a BAR score of ≥18 as a critical cutoff point beyond which liver transplantation would be futile where the probability of survival was significantly reduced. Using a lower cutoff value, on the other hand, would maximise the survival benefit while excluding many candidates from receiving a liver transplant. For example, if a BAR score cutoff value of less than 14 points is used to decide futility, patients with a MELD score of >35 will have a BAR of ≥14, which means that they will be excluded from transplant. Furthermore, using a BAR score of less than 10 points will exclude patients with a MELD score of 26–35 (BAR of 10) from transplantation, except if their ages are ≤40 years, their donor age is <40 years, they have no previous transplant, they do not need pre-transplant life support measures, and the cold ischemic time is ≤6 h. This conflict indicates that the lower cutoff values that achieve a higher survival outcome should not be necessarily adopted to decide candidacy.

The main shortcomings of the current study are the relatively small sample size and its retrospective and single-center nature.

## 5. Conclusions

Finally, we can conclude that the BAR score is a reliable simple scoring system that could accurately predict survival after LDLT. A BAR score of less than 10 points independently predicts post-liver transplantation short- and long-term survival in the Egyptian population. A large-scale multicenter prospective study is warranted to validate our results.

## Figures and Tables

**Table 1:** Demographic, clinical, laboratory, operative, and survival data.

*n* = 146	Mean ± SD, *n* (%)

Demographics	
Age (years)	52.45 *±* 8.54
Sex *n* (%)	
Males	87 (59.6)
Females	59 (40.4)
Etiology of liver disease	
HCV	125 (85.6)
HBV	6 (4.1)
Combined HCV and HBV	3 (2)
NAFLD	5 (3.4)
AIH	3 (2)
Others	4 (2.7)
Clinical data	
BMI (kg/m^2^)	25.47 ± 3.18
Preexisting DM	31 (21.2)
Preexisting HT	9 (6.2)
Life support	20 (13.7)
ICU stay	9.79 ± 3.40
Total hospital stay	24.79 ± 5.65
HCC	37 (25.3)
Laboratory data and scores	
Albumin (gm/dL)	2.58 ± 0.78
Bilirubin (mg/dL)	3.67 ± 5.51
INR	1.40 ± 0.30
Creatinine (mg/dL)	0.98 ± 0.26
AST (IU/L)	66.37 ± 17.50
ALT (IU/L)	40.96 ± 10.79
Hb (gm/dL)	11.06 ± 1.13
WBC (×10³/mm³)	5.00 ± 1.45
Platelets (×10³/mm³)	172.87 ± 127.85
MELD score	14.45 ± 5.0
BAR score	7.58 ± 3.83
Donor age (years)	26.45 ± 6.06
Operative data	
Graft weight (gm)	870.0 ± 15.0
GRWR	1.05 ± 0.15
CIT (minutes)	61.81 ± 25.49
WIT (minutes)	53.01 ± 16.20
TOT (hours)	13.88 ± 3.00
Blood transfusion (units)	5.17 ± 4.64
Survival data	
Three-month survival	130 (89)
One-year survival	115 (78.8)
Five-year survival	105 (72)

BMI, body mass index; ALT, alanine aminotransferase; AST, aspartate aminotransferase; Hb, hemoglobin; WBC, white blood count; HCV, hepatitis C virus; INR, international normalized ratio.

**Table 2:** Univariate and multivariate analysis of variables associated with survival after LDLT.

Variables	3-month survival analysis					1-year survival analysis					5-year survival analysis				
	Survival	Univariate analysis	Cox regression model			Survival	Univariate analysis	Cox regression model			Survival	Univariate analysis	Cox regression model		
	Yes/no 130/16	*p* value	OR	CI (95%)	*p* value	Yes/no 115/31	*p* value	OR	CI (95%)	*p* value	Yes/no 105/41	*p* value	OR	CI (95%)	*p* value

Sex															
Males	78/9	0.291				69/19	0.703				63/24	0.952			
Females	52/7					46/12					42/17				
HCC															
No	101/8	0.540				92/17	0.815				93/16	0.720			
Yes	29/8					23/14					12/25				
WIT															
< 45 min	46/4	0.944				37/9	0.627				31/15	0.132			
≥ 45 min	84/12					78/22					74/26				
GRWR															
0.8–1	68/12	0.888				64/16	0.776				60/20	0.773			
> 1.0	62/4					51/15					45/21				
TOT															
< 12 hour	82/4	0.001^*∗*^	7.34	2.06–26.23	0.002^*∗*^	75/11	0.008^∗^	3.37	1.6–7.2	0.004^*∗*^	69/17	0.009^∗^	2.66	1.42–5.0	0.056
≥ 12 hour	48/12					40/20					36/24				
Blood transfusion															
< 6 units	116/5	0.040^*∗*^	1.31	0.47–3.65	0.601	102/19	0.559				93/28	0.284			
≥ 6 units	14/11					13/12					12/13				
ICU stay															
< 10 days	74/7	0.046^*∗*^	1.12	0.32–3.98	0.860	61/15	0.156				60/21	0.349			
≥ 10 days	56/9					54/16					45/20				
Total hospital stay															
< 1 month	103/15	0.327				27/6	0.719				84/34	0.864			
≥ 1 month	27/1					119/10					19/7				
BAR score															
< 10	115/8	<0.0001^*∗*^	6.35	2.28–17.68	<0.0001^*∗*^	101/22	0.001^∗^	3.21	1.46–7.08	0.001^*∗*^	93/30	0.002^∗^	2.83	1.40–5.7	0.044^*∗*^
≥ 10	15/8					14/9					12/11				

HCC, hepatocellular carcinoma; WIT, warm ischemia time; GRWR, graft recipient weight ratio; TOT, total operative time; ICU, intensive care unit; BAR, balance of risk; ^∗^indicates statistically significant value.

## Data Availability

The data used to support the findings of this study can be acquired from the corresponding author upon request.

## Abbreviations

BAR: Balance of risk
DDLT: Deceased donor liver transplantation
LDLT: Living donor liver transplantation
ROC curve: Receiver operating characteristic curve
AUROC: Area under the receiver operating characteristic curve
OR: Odds ratio
BMI: Body mass index
MELD: Model for end-stage liver disease
D-MELD: Donor age-MELD score
UNOS: United Network for Organ Sharing
DRI: Donor risk index
SOFT: Survival outcomes following liver transplantation
CI: Confidence interval
ALT: Alanine aminotransferase
AST: Aspartate aminotransferase
Hb: Hemoglobin
WBC: White blood count
HCV: Hepatitis C virus
INR: International normalized ratio
HCC: Hepatocellular carcinoma
WIT: Warm ischemia time
GRWR: Graft recipient weight ratio
ICU: Intensive care unit.
